# Stimulus-dependent synchronization in delayed-coupled neuronal networks

**DOI:** 10.1038/srep23471

**Published:** 2016-03-22

**Authors:** Zahra G. Esfahani, Leonardo L. Gollo, Alireza Valizadeh

**Affiliations:** 1 Department of physics, Institute for Advanced Studies in Basic Sciences, Zanjan, Iran; 2 Systems Neuroscience Group, QIMR Berghofer Medical Research Institute, Brisbane, Queensland, Australia; 3 School of Cognitive Sciences, IPM, Niavaran, Tehran, Iran

## Abstract

Time delay is a general feature of all interactions. Although the effects of delayed interaction are often neglected when the intrinsic dynamics is much slower than the coupling delay, they can be crucial otherwise. We show that delayed coupled neuronal networks support transitions between synchronous and asynchronous states when the level of input to the network changes. The level of input determines the oscillation period of neurons and hence whether time-delayed connections are synchronizing or desynchronizing. We find that synchronizing connections lead to synchronous dynamics, whereas desynchronizing connections lead to out-of-phase oscillations in network motifs and to frustrated states with asynchronous dynamics in large networks. Since the impact of a neuronal network to downstream neurons increases when spikes are synchronous, networks with delayed connections can serve as gatekeeper layers mediating the firing transfer to other regions. This mechanism can regulate the opening and closing of communicating channels between cortical layers on demand.

Neuronal spikes constitute a foundation of brain activity yet how neurons and brain regions coordinate their interactions remains as an elephant in the room. Throughout the neocortex, the spiking activity of groups of cells has been found to exhibit various patterns of synchrony, during both spontaneous activity and under sensory stimulation^1^,^2^,^3^,^4^,^5^,^6^,^7^,^8^. Transient synchrony between spiking activity of the neurons has been reported in different sensory tasks^9^ including visual^10^,^11^,^12^, olfactory^13^, and auditory^14^,^15^ as well as in motor system^16^. The functional role of these widespread coherent firing of neurons has been the subject of extensive debates^17^,^18^,^19^,^20^,^21^, and their disturbances have been associated with abnormal brain activity^22^,^23^,^24^,^25^,^26^. It has been hypothesized that a dynamically changing coherent pattern of neuronal activity should ride on top of the anatomical structure and thus provide flexible functional channels of communication, which finds application in selective attention^27^,^28^,^29^,^30^,^31^,^32^ and the binding problem^33^. However, how these structural channels can be effectively controlled to perform specific functions is still unclear.

Since coincidental spikes have greater impact over postsynaptic neurons than otherwise, a possible way to generate the modulation of functional channels is via intermittent synchronization. Characterized by alternation between synchrony and asynchrony, the regime of intermittent synchronization is a signature of systems of oscillators near a bifurcation point (phase transition): Before the onset of complete synchrony this intermittent state can be seen without noise and after onset of synchrony noise can induce intermittent synchrony^34^,^35^. Slow varying parameters and the balance of excitation and inhibition can also make the system switch between synchronous and asynchronous states^36^; neuronal networks with short term plasticity show population bursts intermittent with large periods of asynchronous firing^37^. Here we study the transient patterns of synchrony that appear due to the variable incoming stimuli in neuronal networks coupled by delayed synapses. The neuronal population may represent, for example, a cortical sub-network consisting of neurons with similar receptive fields whose inputs change in the presence of a specific stimulus. We show that the coupling delay plays a crucial role in allowing the network to promptly engage and disengage in a synchronous state depending on the input level even in the absence of noise. In a suitable range of parameters, without fine tuning, an initially incoherent firing of neurons can turn to coherent network oscillation when the mean input is changed—not necessarily increased—through sensory or control input. In the case of a control input, the network can be considered as a controllable channel for the transmission of signals to downstream regions.

The ability of the studied neuronal networks to switch between coherent and incoherent firing, lies on the relation between delay and the oscillatory period^38^,^39^,^40^. It has been shown that two reciprocally coupled neurons can fire in phase if the delays lie in the region where the phase resetting curve (PRC) of the neurons have negative slope^41^, otherwise their firing is in antiphase^42^,^43^,^44^. In large networks where neurons are connected to several other neurons, in-phase firing remains stable where instead of antiphase state, multiple competing metastable states appear. This phenomenon speaks to geometric *frustration* in condensed matter physics where a plenitude of distinct ground states are ensued by the lattice structure as, for example, in Ising system with triangular lattices and anti-ferromagnet interactions^45^. Although the rich dynamics of frustrated systems have been shown to increase the dynamical repertoire of a network^46^, induce metastability^47^,^48^, and multistability^49^, the potential for a flexible system to switch between frustrated and non-frustrated systems as a function of the external input has remained unexplored.

In a delay-coupled system, the connections can be synchronizing (ferromagnet) or desynchronizing (anti-ferromagnet) depending on the ratio of the delay and the mean inter-spike intervals. Changing the input changes the inter-spike intervals, and the synchronization. We characterize the (de)synchronizing properties of 2-neuron, 3-neuron motifs, and large networks. We demonstrate that large delayed-coupled networks can switch between desynchronized (frustrated) states and a synchronized (non-frustrated) state depending on the external driving. This mechanism can be effective for a prompt control of the oscillatory dynamics of networks, endowing them with the ability to selectively activate neurons (and regions) downstream along the anatomical pathway. Such gatekeeping mechanism taking place in a plausible time scale of a few hundreds of milliseconds might be a cornerstone of brain functioning and perhaps one of the most fascinating implications of coupling delay in the brain.

## Material and Methods

### Simplified pulse-coupled neuronal oscillators

Our model consists of *N* excitatory neurons in a network with random or all-to-all connectivity. The evolution of the state vector of the neuron *X*_*i*_ can be described by the system of coupled ordinary differential equations


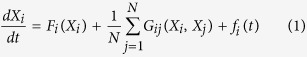


for *i* = 1, …, *N*. Here *F*_*i*_(*X*_*i*_) describes the autonomous state of the *i*th oscillator, *G*_*ij*_(*X*_*i*_, *X*_*j*_) denotes the interactions of the *i*th neuron with the other neurons in the network, and *f*_*i*_(*t*) is the external drive. The external input to each neuron comprises a constant current but similar results are also obtained when the current is chosen from a narrow normal distribution, and an independent Gaussian white noise. The currents are supra-threshold so that the neurons are in the oscillatory regime and their state can be described by the phase variable. Assuming connections are weak and the noise is of small amplitude, phase representation remains valid for the neurons in the network. We perform the phase reduction of Eq. 1, defining the phase *ϕ* for *X* near a limit cycle. The equation describing the reduced dynamics is


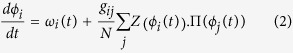


where *ω*_*i*_ is the intrinsic firing rate of the neurons which is controlled by the external input. The vector *Z*(ϕ) is the phase sensitivity of the oscillators which characterizes the response of the phase oscillator to external perturbations. *g*_*ij*_Π(ϕ) is the contribution of neuron *j* to the field exerted on neuron *i*. The neurons’ spiking activity is recorded as Dirac’s delta functions 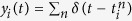 where 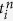 is the time of the *n*^*th*^ spike of the neuron *i*, defined as the instant at which the phase of neuron crosses multiples of 2*π*.

**Figure 1:**
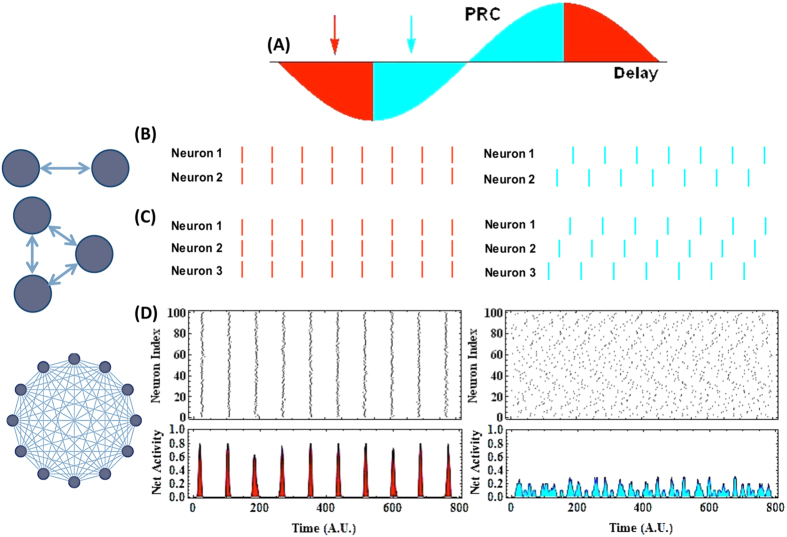
Communication delay determines the collective state of neuronal networks. (**A**) Slope of phase resetting curve (PRC) determines the nature of synaptic connections. If delay (normalized by period of firing) is chosen from the region over which PRC has a negative slope (red filled), the connection is synchronizing, and from the region of positive slope of PRC (cyan filled), the connection is desynchronizing. In (**B**–**D**) the state of the coupled neurons is depicted for two delay values shown with arrows in (**A**); *τ*/*T* = 1/6 leads to synchronizing connections (red arrow, left panels) and *τ*/*T* = 1/3 to desynchronizing connections (cyan arrow, right panels). (**B**) Two-neuron motif with reciprocal connections: inphase (left) and anti-phase firing (right) occur depending on the delay time. (**C**) Fully connected three-neuron motif: synchronizing connections lead to synchronous firing of the three neurons (left), but other stable states can emerge with desynchronizing connections; one of them is shown when neurons fire with 2*π*/3 phase lag. (**D**) Raster plot (top) and network activity (bottom) are shown for a fully connected network with 100 neurons. For synchronizing connections, the synchronous state maintains stability in large network which leads to oscillatory network activity (left panels, 〈*r*〉_*t*_ = 0.79). For desynchronizing connections (right) asynchronous firing of the neurons leads to small amplitude noisy network activity (right panels, 〈*r*〉_*t*_ = 0.07). In (**B**,**C**) the noise amplitude is zero and the neurons are identical for the sake of illustration. In (**D**) independent Gaussian white noises with zero mean and intensity *D* = 0.05 is added to the input of the neurons, described by the phase approximation Eq. 3.

We further simplified the model by assuming the neuronal oscillators are pulse coupled. This form of pulse-coupling idealizes the fact that in diverse biological systems, such as populations or networks of spiking neurons in the brain, units interact by stereotyped signals whose time courses are short compared to the mean inter-spike intervals^50^,^51^,^52^. Dynamical system in this case is described by


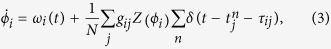


where *g*_*ij*_ is the strength of synaptic connection and is chosen equal to 1 unless stated otherwise, *τ*_*ij*_ is the transmission delay time from presynaptic to postsynaptic neuron which is chosen from a narrow Gaussian distribution with variable mean and width *σ* = 0.1. In the simulation with phase oscillators (Fig. 1 and Supplementary Fig. S5) all the parameters and the time are unit-less and the mean period of firing is 2*π*.

### Synchronization order parameter

The network activity is defined as 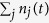, where *n*_*j*_(*t*) is the number of spikes of neuron *j* in the network in a short sliding interval. Synchronous spiking of the neurons leads to oscillatory network activity with large amplitude where incoherent spiking leads to noisy fluctuations of network activity with small amplitude^53^. To quantify synchrony we have also used “Kuramoto order parameter”


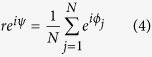


where *ϕ*_*j*_ is the phase of the *j*th oscillator and 0 ≤ *r*(*t*) ≤ 1 measures the phase coherence of the population and *ψ*(*t*) is the collective phase of the population. The average value of the order parameter over time 〈*r*〉_*t*_ is a measure of synchrony in the network. 〈*r*〉_*t*_ = 1 means fully synchronized state and 〈*r*〉_*t*_ = 0 represent the state in which the phase of oscillators are distributed randomly.

### Hodgkin-Huxley neuron

In our simulations we have also used the Hodgkin-Huxley (HH) model^54^, which is described by a set of four variables *X* = (*V*, *m*, *n*, *h*), where *V* is the membrane potential, *m* and *h* are the activation and inactivation variables of the sodium current and *n* is the activation variable of the potassium current. The equations governing the evolution of the variables are


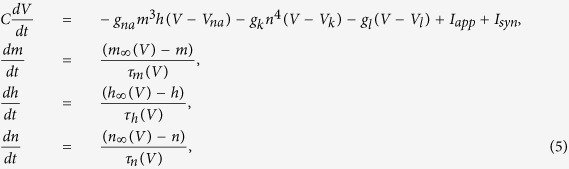


where *I*_*app*_ and *I*_*syn*_ are the external and the synaptic currents, respectively. The parameters *g*_*na*_, *g*_*k*_ and *g*_*l*_ are the corresponding reversal potentials and *C* is the capacitance per surface unit. We use the typical parameter values: *V*_*na*_ = 50 *mV*, *V*_*k*_ = −77 *mV*, *V*_*l*_ = −54.4 *mV*, *g*_*na*_ = 120 *mS*/*cm*^2^, *g*_*k*_ = 36 *mS*/*cm*^2^, *g*_*l*_ = 0.3 *mS*/*cm*^2^ and *C* = 1 *μF*/*cm*^2^. The functions *m*_∞_(*V*), *h*_∞_(*V*) and *n*_∞_(*V*) and the characteristic times *τ*_*m*_, *τ*_*n*_, *τ*_*h*_ (in milliseconds) are given by: 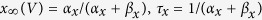 with *x* = *m*, *n*, *h* and *α*_*m*_ = 0.1(*V* + 40)/(1 − *exp*[(−*V* − 40)/10], *β*_*m*_ = 4 *exp*[(−*V* − 65)/18], *α*_*h*_ = 0.07 *exp*[(−*V* − 65)/20], *β*_*h*_ = 1/(1 + *exp*[(−*V* − 35)/10]), *α*_*n*_ = 0.01(*V* + 55)/(1 − *exp*[(−*V* − 55)/10]), *β*_*n*_ = 0.125 *exp*[(−*V* − 65)/80]. Let us recall that for small values of *I*_*app*_ the system reaches a stable fixed point (*V* = −65 *mV* for *I*_*app*_ = 0). The transition from resting to spiking regime is mediated by a subcritical Hopf bifurcation at the critical value of input current *I* = 9.2 *μA*/*cm*^2^. The synaptic current *I*_*syn*_ is modeled by


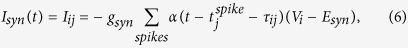


where the *α* function


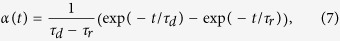


describes the postsynaptic conductance time course after each spiking of presynaptic neuron at time 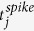. The time delay *τ*_*ij*_ is the time needed for the transmission of signals from the pre- to the postsynaptic neuron, in this case from *j*th to *i*th neuron.

We used delays varying from 0 to 14.6 ms (a period of oscillations for *I*_*app*_ = 10 *μA*). The reversal potential of the synapse is *E*_*syn*_ and *τ*_*r*_ and *τ*_*d*_ determine the rise time and decay time of the synaptic response, respectively. We set *E*_*syn*_ = 30 *mV* to model excitatory synapses, and *τ*_*r*_ = 0.2 *ms* and *τ*_*d*_ = 3 *ms*.

## Results

In the weak-coupling regime, once the PRC of the neurons is known, the ratio of the delay time to the period of firing of neurons determines whether the connection is synchronizing (red area of Fig. 1) or desynchronizing (blue area of Fig. 1)^42^,^55^. The in-phase (or synchronizing) solution is stable for two reciprocally connected neurons with excitatory synapses when the slope of the PRC is negative; otherwise the antiphase solution is stable (see Fig. 1A,B and Supplementary Material).

Representative results for two canonical type-II neurons for which the PRC can be approximated by −sin (*θ*)^56^ as shown in Fig. 1B: Neurons fire synchronously or in the opposite phase when the ratio of communication delay to the period of firing lies respectively in the region of negative or positive slopes of the PRC (Fig. 1B). In a motif of three mutually connected neurons, in-phase solution is also globally stable when the PRC has a negative slope. However, the system shows *multistability* when the PRC has a positive slope. Performing a linear stability analysis (see Supplementary Material, Sec. S1), we find (i) one class of stable states in which two neurons fire in phase and they are in antiphase with respect to the third neuron (permutations of (0, *π*, *π*) state), and (ii) another class the neural phases which is evenly distributed over the cycle with ±2*π*/3 phase differences, which is illustrated in Fig. 1C. Our numerical experiments show that despite the linear stability, the (0, *π*, *π*) state has a smaller basin of attraction and therefore most trials with random initial conditions exhibit the (2*π*/3, 2*π*/3, 2*π*/3) state. In large networks with *N* ≫ 1, the same condition determines the stability of the synchronous solution, and the time delays in which the PRC has a negative slope lead to coherent synchronous firing of neurons, causing oscillatory network activity (Fig. 1D, left). Other values of delay which would result in antiphase firing of the two-neuron motif, here lead to incoherent firing of neurons. Asynchronous activity of neurons leads to irregular and noisy network activity (Fig. 1D, right) with a small value of the synchronization order parameter (Figs S3A and S4A).

**Figure 2:**
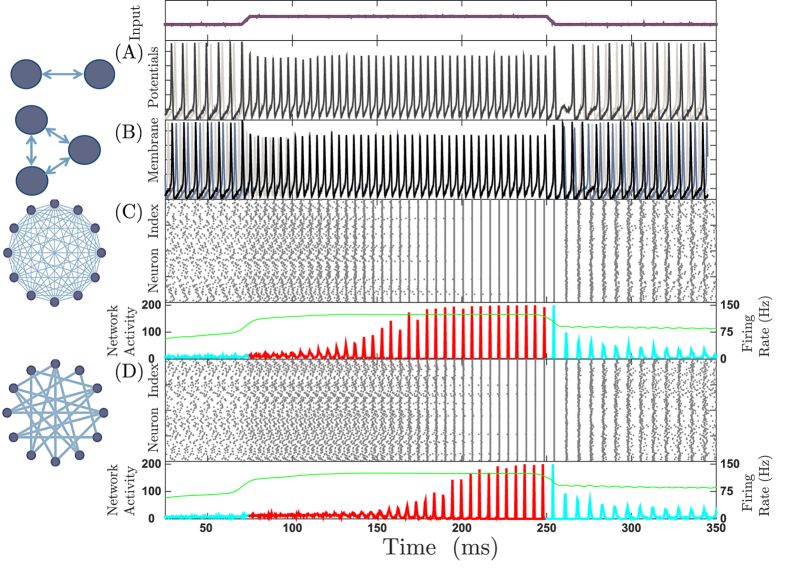
Input level determines the collective behavior of the neuronal networks with delayed interactions. Changing the level of input (top panel) may lead to changes in the synchronization. In (**A**,**B**) Two- and three-neurons motif: Anti-phase and out-of-phase firing changes to in-phase firing when the input increases. (**C**,**D**): Large networks (*N* = 500) with the same input variation leads to coherent firing of the neurons for all-to all and random networks with connection probability *p* = 0.5 respectively. Top panel: Raster plot of 200 randomly selected neurons; bottom panel: Firing rate. The green line shows the firing rate of network which is computed using a Gaussian sliding window with *σ* = 25 *ms*. We used HH neurons connected through excitatory chemical synapses with connection delay of *τ* = 9 *ms* when *I*_*app*_ is increased from 10 *μA* to 20 *μA*, as described in Materials and Methods. For more details Supplementary Fig. S2 and Table S1 are available.

In a real neuronal network delays are essentially fixed (in the time scale of interest, see however counterexamples in^57^), but the inter-spike intervals can evolve and play a much more flexible role in response to variable external inputs. As noted above, the ratio of the delay time to the period of the firing determines whether the coupling is synchronizing or desynchronizing. Hence, changing the level of input to the neurons can convert a synchronizing connection to a desynchronizing one and vice versa. Reflecting that desynchronizing couplings in 2-neuron (3-neuron) motifs lead to antiphase (out-of-phase) firing, Fig. 2A(B) shows how a variation on the external input in two (three) HH-neuron motif changes the dynamics from in-phase firing to antiphase (out-of-phase) and vice versa. Since desynchronizing connections in large networks (*N* ≫ 1) lead to incoherent activity, similar changes in the input result in the transitions between synchronous and asynchronous collective dynamics, which is evident in the network activity as transitions between low amplitude noisy activity and large amplitude oscillatory dynamics occur (Fig. 2C,D).

Notably, these transitions between the different dynamic states occur in a short time interval of ~100 milliseconds. Such a response of the network to the varying inputs is not dependent on the all-to-all connectivity of the network and analogous transitions are observed in random networks (Fig. 2D). The main difference between the two network topologies is that the transition to the synchronous state occurs faster in fully connected than sparser random networks (see Supplementary Figs S3 and S4). The presence of noise in the form of stochastic input or inhomogeneity in the intrinsic firing frequencies decreases the coherence of the oscillations. Nonetheless the switching between incoherent and coherent states can be perceptibly identified for small or intermediate levels of stochastic inputs or disorder (see Supplementary Fig. S5). It is worth mentioning that the transition from asynchrony to synchrony does not necessarily occur when the input current is increased. As noted above when the delay-to-period ratio changes such that it passes from a region of positive to negative slope of the PRC, the system switches to a synchronous state; depending on the functional form of the PRC this transition can also be seen with a decrease in the external current.

**Figure 3:**
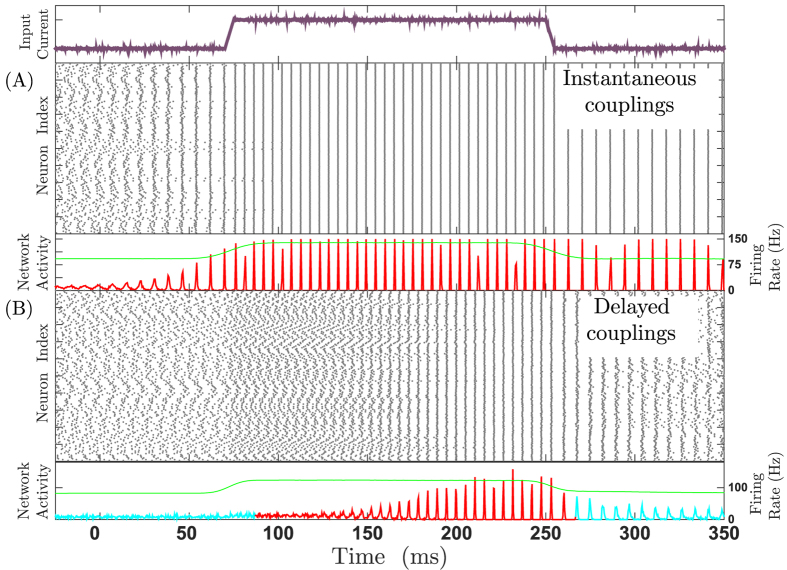
Role of delay in stimulus dependent collective dynamics. Panels of input current, raster plots and firing rate time trace depicted as in the previous Fig. 2. In the networks with instantaneous connections change in input only changes the firing rate and not the state of the network (**A**), while in a network with delayed connections the change in the input level changes the collective dynamics from asynchrony to synchrony and vice versa (**B**). For more details Supplementary Fig. S2 is available.

**Figure 4:**
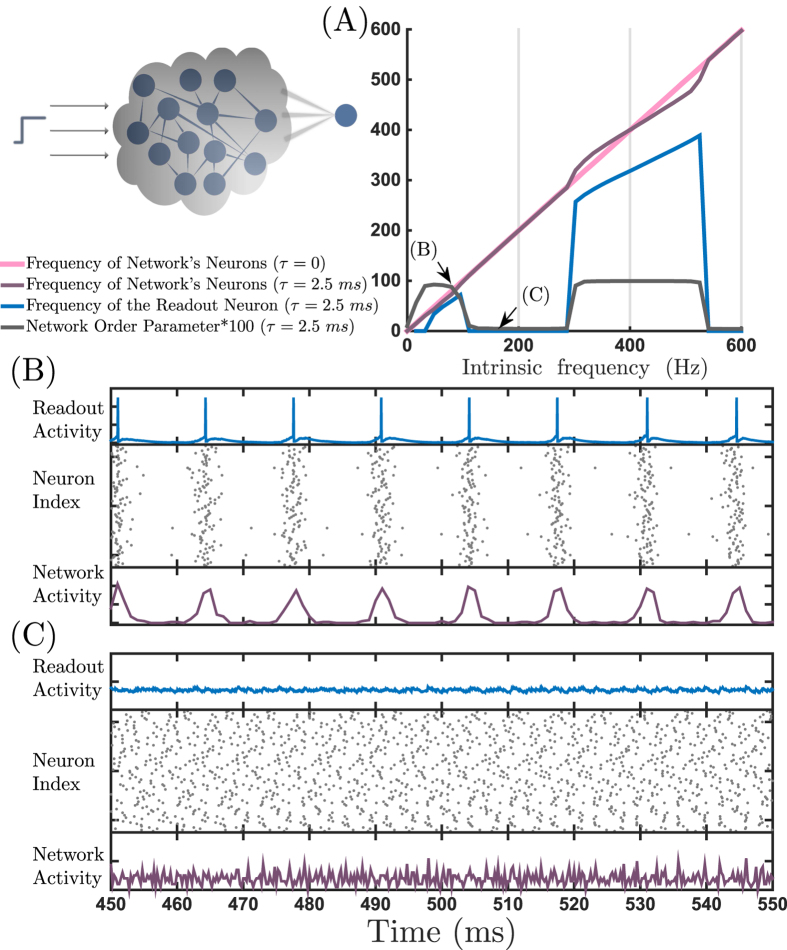
Response of the readout neuron to indirect stimulus. (**A**) The pink and purple lines show the mean frequency of the network versus the input currents for instantaneous (without delay) and delayed connections, respectively. The gray curve demonstrates the order parameter of the network and the blue curve represents the response of the readout neuron to the network activity. (**B**,**C**) show the voltage of the readout neuron (top), raster plot of the spiking activity of the network (middle) and the network activity (bottom) for two different amounts of input current which lead to coherent and incoherent activity of the network, respectively.

Synaptic delays are essential to allow transitions in the oscillatory dynamics of the network. Figure 3 compares the response of random networks to a change in the input in the presence of delayed interactions with a network of instantaneous interactions (no delay). Networks with delayed interactions switch their firing pattern between coherent and incoherent states depending on the input level (Fig. 3B). In contrast, networks with instantaneous connections synchronize for all input levels (as long as connections are synchronizing), and varying the input level changes the oscillatory frequency and the firing rate of the network but does not desynchronize it (Fig. 3A). As a result, only networks with delayed connections exhibit synchronization control as a function of the input level. Another feature of such stimulus-dependent synchronization is that networks oscillate solely at specific ranges of frequency Fig. 4.

Since coincident spikes play a crucial role in the reliable propagation of spikes, the selective synchronization property of delayed networks gives rise to a gatekeeping function of the network: activating or not a transmission channel and neurons in a receiver layer. It is well-known that downstream neurons are affected more profoundly in case of synchronous spiking of a neuronal population^58^. This can reveal a unique feature of delayed connections in building a dynamic channel of communication which can be opened and closed depending on whether the input leads to synchronous or an incoherent state. This mechanism is demonstrated in Fig. 4. A readout neuron, which receives weak synaptic inputs from neurons of the population, can only spike if it receives several input pulses almost simultaneously. Therefore, the output neuron goes On and Off depending on the level of the input to the population (Fig. 4). Note that without the mediating population, or with a population with instantaneous synchronizing connections, the output neuron would respond to any level of the input once it is supra-threshold (Fig. 4).

## Discussion

A flexible communication structure between neuronal groups is a requirement for cognitive flexibility^17^. However, despite few attempts^59^,^60^, the mechanism of dynamic modulation of neuronal interactions (or the selection of engaged and disengaged interactive channels) remains largely mysterious. Here we put forward a mechanism that allows a network to transfer oscillatory signals at specific frequency ranges, selectively activating output neurons. According to this gatekeeping mechanism, neuronal groups may or may not propagate their activation to a next level depending on the input levels, which determines whether their firing is synchronized or not.

The key ingredient of neuronal networks to generate a controllable communicating channel is conduction delay. Far from being a nuisance, the presence of delay can massively enrich the dynamics providing metastability^61^, multistability^49^, clustering^39^, frustration^46^,^62^, and increased synchronization^63^ to the systems. Coupling delay is a prominent feature of large-scale brain connectivity, and therefore any proposed model or mechanism of brain functioning must take it into account, see e.g.^64^,^65^,^66^. Not surprisingly, delay is also a crucial element to reproduce resting brain fluctuations^67^,^68^,^69^. In addition, the complex behavior of delay-coupled systems can be applied to encrypted communication, fast random bit generator^70^, as well as to bioinspired information processing^71^,^72^.

Here we report a novel function of delay that allows for neuronal networks to be controlled, which is fast and reliable and thus could be explored in bioinspired computing methods. Previous hypothesis for the prompt control of engagement and disengagement of coherent dynamics between two regions require changes in the balance of excitation/inhibition of cortical regions^36^, or a thalamic relay^59^. On the other hand, as presented here, delayed-coupled networks enable the *reentrant activity*^73^ between two cortical regions to be modulated and controlled parsimoniously by the level of input. Moreover, since the oscillatory band of transmission is a property of the network, each directed channel can operate at a particular frequency. Hence, this mechanism supports, for instance, the proposal that feedforward signals are transmitted in theta and gamma rhythms whereas feedback signals are transmitted in beta rhythms in visual areas^74^.

In the presence of delay in the interaction between two identical limit cycle oscillators with a given phase resetting curve, in-phase or anti-phase oscillations can emerge depending on the ratio of the delay to the period of the oscillation^43^ and the type (excitatory/inhibitory) of connections^75^,^76^. This relation can be explored to define synchronizing and desynchronizing connections. When connections between pairs of oscillators are synchronizing, synchronous oscillations occur; otherwise, they exhibit out-of-phase oscillations. In large networks, where each oscillator is connected to several other oscillators in the network, the inconsistency of out-of-phase oscillations induces incoherent firing. This phenomenon echoes the so called frustration phenomenon in condensed matter physics^45^, and frustrated states correspond to an asynchronous state.

Frustration in condensed matter physics refers to a phenomenon where components of the system experience multiple competing interactions with other components so that decreasing the energy of one of the links increases the energy of other links, and the ground state (the state of minimum energy) cannot be easily deduced from the properties of the system. For example when three magnetic spins with anti-ferromagnetic interactions are located on the corners of a triangle, the energy of each link is minimized if the corresponding pair of spins is aligned in opposite directions. Once two spins are anti-parallel, the third one experiences frustration since there is no favorable direction to simultaneously minimize energy of its interaction with two other components. In this case several arrangements may have the same energy and new stable states can emerge. The behavior of neurons and cortical areas have also been associated with frustrated states that enhances the metastability or multistability of the system^46^,^47^,^48^,^49^. In this case, 3-node circuits with desynchronizing coupling correspond to a minimal frustrated system with multiple locally stable dynamical states (two states with phase difference of ±2*π*/3, and three states with two in-phase neurons and the third neuron in antiphase). In large frustrated networks the firing complexity is greatly increased, and the network activity is compatible with an incoherent state.

In this study we have shown that a network of neuronal oscillators with delayed connections can switch between synchronous network oscillations and incoherent firing activity, when the input to the network changes. Different aspects of synchronization of neuronal oscillators have been extensively studied in recent decades and the possible role of the type of synapses^77^,^78^, synaptic plasticity^79^,^80^,^81^,^82^,^83^, and effect of transmission delays^84^,^85^ have been explored. It is well-known that the role of the synaptic connections in synchronizing the neuronal oscillators can be identified once the phase resetting curve of the neurons, inter-spike intervals and the time delay are known^55^,^86^,^87^. In particular, the synchronizing effect of the connections changes when either the time delay or the inter-spike interval changes. In realistic neuronal networks, time delays can be considered as fixed values but the spiking frequency of the neurons varies due to the variations in the mean input they receive in different cognitive and sensory tasks. Our results show that neuronal networks in the presence of delayed interactions may change their collective behavior (leading to transient synchrony) in response to a modulatory input. Such transient network oscillations have been reported in different sensory systems at the onset of exposure of the sensory input to the system^10^,^11^,^13^,^14^. It is worth noting that in a network with instantaneous connections, when delay is ignored, the collective state of the network does not depend on the input level and changing input only affects the firing rate of the network (see Fig. 4).

This study reveals a possible role of the delay in the collective behavior of neuronal systems. We provide a proof of principle for a stimulus-dependent synchronization in neuronal systems. Although we mainly focus on simple delayed-coupled oscillatory systems with small heterogeneity in the time delay, we extended its plausibility to more realistic scenarios with inhomogeneous firing rate and in the presence of noise. We have also used a few simplifying assumptions whose role are yet to be elucidated. These assumptions, including considering neurons as oscillators, homogeneous delays and pure excitatory network can be relaxed in future studies to explore, for example, the role of non-homogeneous delay in the dynamics of balanced networks of excitatory and inhibitory neurons.

## Additional Information

**How to cite this article**: Esfahani, Z. G. *et al.* Stimulus-dependent synchronization in delayed-coupled neuronal networks. *Sci. Rep.* **6**, 23471; doi: 10.1038/srep23471 (2016).

## Supplementary Material

Supplementary Information
