# Supplementary material for: Stimulus-dependent synchronization in delayed-coupled neuronal networks
Source: Sci Rep. 2016 Mar 22;6:23471. doi: 10.1038/srep23471 (PMC4802300; doi:10.1038/srep23471)
Supplement: Supplementary Information [file srep23471-s1.pdf]

Supplementary Material for "Stimulus-dependent synchronization in delayed-coupled neuronal networks"

Zahra G Esfahani[1], Leonardo L Gollo[2] and Alireza Valizadeh[1,3]

[1]Department of physics, Institute for Advanced Studies in Basic Sciences, Zanjan, Iran
[2]Systems Neuroscience Group, QIMR Berghofer Medical Research Institute, Brisbane, Queensland, Australia
[3]School of Cognitive Sciences, IPM, Niavaran, Tehran, Iran

January 31, 2016

Theoretical background

A two-neuron network motif, reciprocally connected through delayed pulsatile interactions, can be described by Eq. 3 with $j = 1, 2$. We construct a map by recording the phase of the neurons at the instant of firings of the first neuron (see Fig. S1) [1, 2]. Assuming the neurons have the same firing rate ω, we have

$$\begin{aligned}\phi_1(t_{n+1}) &= \phi_1(t_n) + \omega T + g_{12} Z(\omega\tau_{12} + \Delta\phi_n),\\ \phi_2(t_{n+1}) &= \phi_2(t_n) + \omega T + g_{21} Z(\omega\tau_{21} - \Delta\phi_n).\end{aligned} \tag{1}$$

where T is the period of firing of the neurons and $\Delta\phi_n = \phi_1(t_n) - \phi_2(t_n)$ is the phase difference of the neurons at the instant of nth spike of the neuron 1. Subtracting the above equations gives the map for the phase lag $\Delta\phi_n$ and its fixed point determines the phase lag in the locked state. In a homogeneous system with identical neurons and synapses ($\omega_1 = \omega_2$, $g_{12} = g_{21}$ and $\tau_{12} = \tau_{21}$), fixed points are the solutions of $Z(\omega\tau + \Delta\phi) = Z(\omega\tau - \Delta\phi)$. Having in mind that $Z(\theta)$ is a 2π periodic function of θ, the latter equation has two solutions $\Delta\phi = 0$ and π. Linearizing the equation for the phase lag around these solutions gives the stability condition. The inphase mode is stable for $Z'(\omega\tau) < 0$ and the anti-phase mode stability condition is $Z'(\omega\tau + \pi) < 0$. For canonical type-II oscillators with $Z(\theta) = -\sin(\theta)$, for example, for $\omega\tau < \frac{\pi}{2}$ and $\omega\tau > \frac{3\pi}{2}$ the connections are *synchronizing* and the synchronous state is stable. For $\frac{\pi}{2} < \omega\tau < \frac{3\pi}{2}$ the connections are *desynchronizing* and the neurons fire in antiphase. Finally, for $\omega\tau = \frac{\pi}{2}$ and $\omega\tau = \frac{3\pi}{2}$, the phase lag depends on the initial condition and all phase lags are neutrally stable. Figure. 1 shows phase lag in the steady state for canonical type-II neurons for two different values of the oscillators' frequency. Note that since $\omega\tau$ determines the stability condition, changing the frequency of the oscillators can change the nature of the connections and hence the state of the system. For the fully connected three-neuron motif, a similar map can be constructed

$$\begin{aligned}\phi_1(t_{n+1}) &= \phi_1(t_n) + \omega T + g_{12} Z(\omega\tau_{12} + \Delta\phi_n) + g_{13} Z(\omega\tau_{13} + (\Delta\phi_n - \Delta\phi'_n)),\\ \phi_2(t_{n+1}) &= \phi_2(t_n) + \omega T + g_{21} Z(\omega\tau_{21} - \Delta\phi_n) + g_{23} Z(\omega\tau_{23} - \Delta\phi'_n),\\ \phi_3(t_{n+1}) &= \phi_3(t_n) + \omega T + g_{31} Z(\omega\tau_{31} - (\Delta\phi_n - \Delta\phi'_n)) + g_{32} Z(\omega\tau_{32} - \Delta\phi'_n).\end{aligned} \tag{2}$$

where T is the period of firing of the neurons and $\Delta\phi_n = \phi_1(t_n) - \phi_2(t_n)$ and $\Delta\phi'_n = \phi_2(t_n) - \phi_3(t_n)$. For $Z(\theta) = -\sin(\theta)$, the fix point of the equations 2 is determined by $sin(\Delta\phi_n) = sin(\Delta\phi'_n)$, which leads to $\Delta\phi_n = \Delta\phi'_n$ and $\Delta\phi_n = \pi - \Delta\phi'_n$. With $\Delta\phi_n = \Delta\phi'_n$ we have two solutions $\Delta\phi_n = \frac{2\pi}{3}$ and $\Delta\phi_n = 0$ which are the evenly spaced phases $(2\pi/3, 2\pi/3, 2\pi/3)$ state and inphase $(0, 0, 0)$ state, respectively; and with $\Delta\phi_n = \pi - \Delta\phi'_n$ we have $\Delta\phi_n = 0$ and $\Delta\phi_n = \pi$ both representing two inphase neurons which are antiphase with the third neuron, $(0, \pi, \pi)$ state.
The linear stability analysis shows that $\Delta\phi_n = \Delta\phi'_n = 0$ is stable when the connections are synchronizing $\lambda = cos(\omega\tau) > 0$, and the two other states $\Delta\phi_n = \Delta\phi'_n = 2\frac{\pi}{3}$ and $\Delta\phi_n = 0, \Delta\phi'_n = \pi$ are stable when connections are desynchronizing $\lambda = cos(\omega\tau) < 0$.

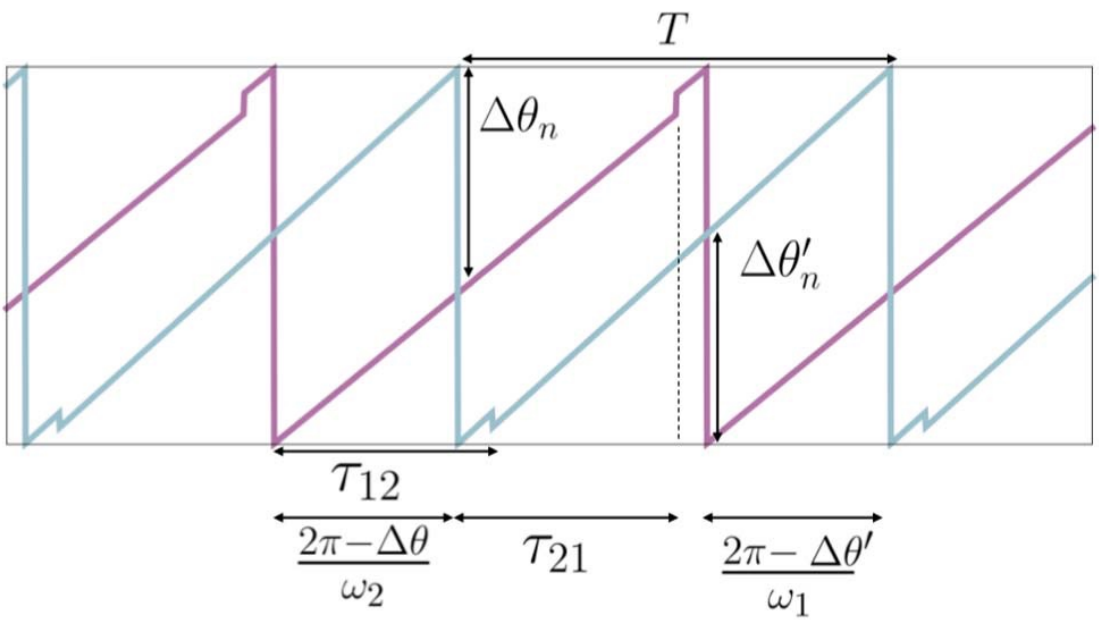

Figure S1: **Time evolution of coupled phase oscillators.** Schematic map of phase versus time used for analytic investigation. The evolution of the averaged phase of two neurons is shown by solid navy and violet lines, respectively. T is period of firing in phase locked state, τ_{ij} is the time delay, and ωs are the intrinsic frequency of oscillators. Dynamical variable of the map is the phase difference of the two neurons at the time of nth spike of the first neuron, depicted as $\Delta\theta_n$.

For a network of N interconnected neurons, the Poincaré map reads

$$\phi_i(t_{n+1}) = \phi_i(t_n) + \omega_i T + \sum_j g_{ij} Z(\omega_i \tau_{ij} - (\phi_i(t_n) - \phi_j(t_n))), \tag{3}$$

where $i = 1, 2, ..., N$. Given a homogeneous network with identical neurons, zero-lag synchronization is a solution of the generalized equation 3, and the stability condition for the synchronous solution is reduced to $Z'(\omega\tau) < 0$. As a result, we can conclude that the stability of zero-lag synchrony of networks with homogeneous degree (e.g. all-to-all networks) is independent from the coupling constant g and the size of the network N.

Changing the synchronization properties of the connections through changes in the input current

To illustrate the effects of a variable synchronization over the network we consider one example of variation of the injected current. When the level of injected current to the network increases from I_1 to I_2 ($I_1 < I_2$), the intrinsic frequency of oscillators increases from ν_1 to ν_2, and the inter-spike interval of oscillations reduces from T_1 to T_2 ($T_1 > T_2$). Figure S2 shows how with a fixed delay the property of the connection changes with the period. If the delay τ lies in the ranges shaded by gray, the connection switches from synchronizing to desynchronizing and vice versa. Table S1 demonstrates the ranges of the delay (in terms of T_1 and T_2) with the corresponding change of the property for a type-II oscillator with canonical form of PRC. As Fig. S2 and table S1 show, a transition to synchrony occurs for $3\frac{T_2}{4} < \tau < 3\frac{T_1}{4}$, and a transition to asynchrony occurs for $\frac{T_2}{4} < \tau < \frac{T_1}{4}$.

Owing to the slowly varying F-I curve (Frequency-Injected current curve) of the Hodgkin-Huxley neurons, varying the input current from 10 μA to 20 μA, the oscillatory frequency rises from 68.6 Hz to 86.9 Hz. Therefore, the oscillatory period reduces from 14.6 ms to 11.5 ms. In this example the delay interval that leads to a transition from a desynchronized to a synchronized state is 8.6 $ms < \tau <$ 10.9 ms, and the delay interval that leads to a transition from a synchronized to a desynchronized state is 2.8 $ms < \tau <$ 3.6 ms. Such range of short delays of about 3 ms shows that this transition could play

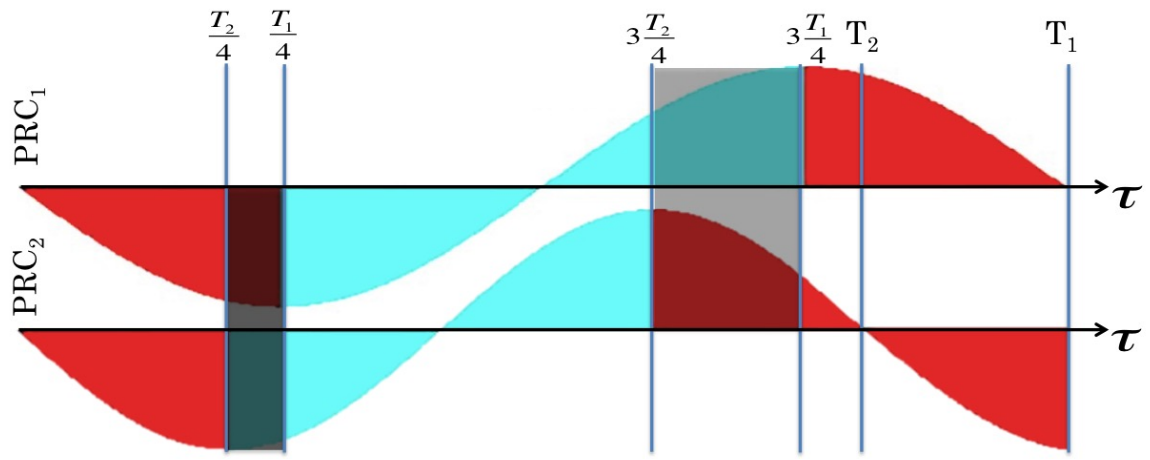

Figure S2: **Connection's properties for two different amounts of injected current.** The oscillatory period is shorter for larger external input. For a fixed interaction delay the final state of synchronization depends on the amount of external input, or the period of the PRCs. Gray shaded areas show the delay intervals in which increasing the external current from I_1 (PRC_1) to I_2 (PRC_2), changes the final state of oscillations from synchronized to desynchronized and vise versa.

Delay	Transition
$\tau < \frac{T_2}{4}$	*Sync* $\longrightarrow$ *Sync*
$\frac{T_2}{4} < \tau < \frac{T_1}{4}$	*Sync* $\longrightarrow$ *Desync*
$\frac{T_1}{4} < \tau < 3\frac{T_2}{4}$	*Desync* $\longrightarrow$ *Desync*
$3\frac{T_2}{4} < \tau < 3\frac{T_1}{4}$	*Desync* $\longrightarrow$ *Sync*
$3\frac{T_1}{4} < \tau < 5\frac{T_2}{4}$	*Sync* $\longrightarrow$ *Sync*
$5\frac{T_2}{4} < \tau < T1$	*Sync* $\longrightarrow$ *Desync*

Table S1: **State transition for different delay intervals.**

an important role for cortical oscillations at gamma band. Note that in a biologically realistic cases, the changes in the firing rates which cause transitions between synchrony and asynchrony, are determined by the form of PRC which might change their slope several times during the whole period, namely earlier in the period (see e.g., [3]), which makes it possible that transitions occur in smaller values of firing rates even with delay times of few milliseconds.

Time-scale of transition to synchrony

Any feasible control mechanism for the opening and closing of communicating channels must operate in a fast time scale that is compatible with the changes between metastable states. As shown in Figs. S3 and S4, the proposed mechanism of engagement and disengagement of synchronization occurs in the order of hundreds of milliseconds for different network sizes, in-degree values and synaptic strengths.

Robustness against noise and heterogeneity

To check how the results are robust against different sources of noise we present the outcome of changing level of the stimulus in presence of stochastic input or quenched noise as the inhomogeneity of firing

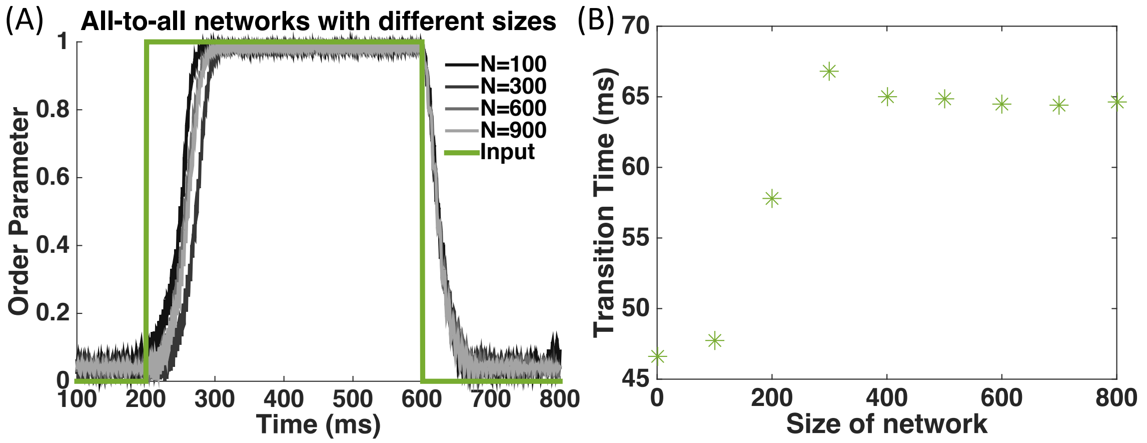

Figure S3: **Transition to synchrony in all-to-all networks with different sizes.** (A) Synchronization order parameter of all-to-all networks versus time. The green line depicts the time-dependent input. The transition time, chosen as the time to reach 50% of the maximum order parameter, is shown in (B) for different network sizes. For large enough networks ($N > 100$), the transition time is independent of the network size.

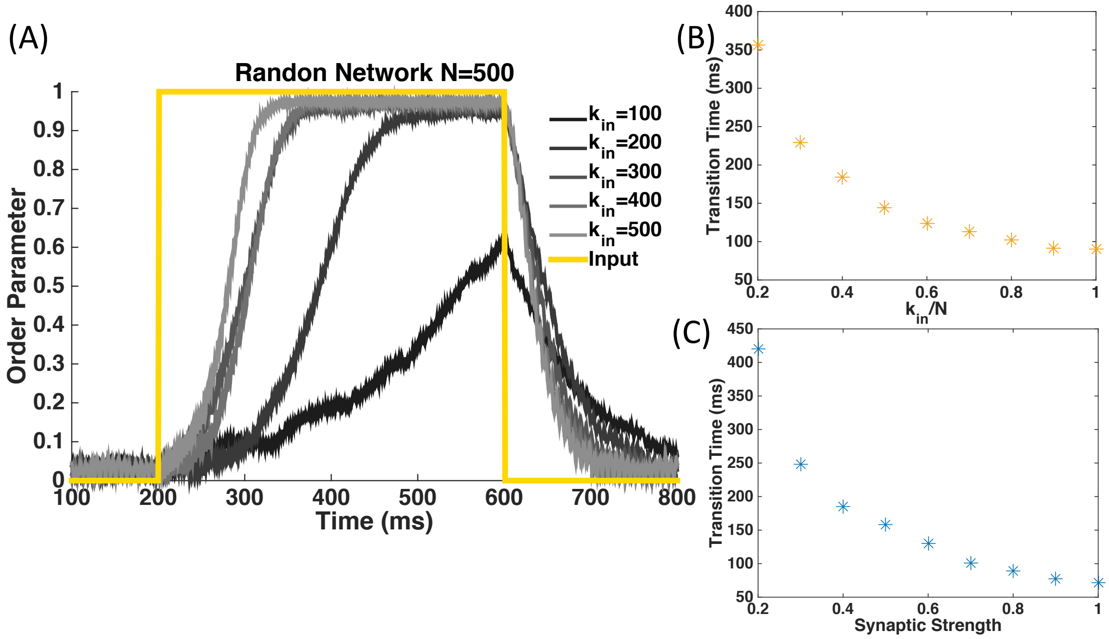

Figure S4: **Transition to synchrony in random networks.** (A) Synchronization order parameter of random networks with different mean in-degree versus time. The orange line depicts the time-dependent input. In (B) and (C) the time needed to reach 50% of maximum order parameter is shown for different mean in-degrees and different synaptic strengths, respectively. The larger is the in-degree or the synaptic strength, the faster is the transition.

frequencies, resulted from imposing different levels of input to the neurons. In general, both noise and inhomogeneity oppose collective coherence and decrease the level of the order in the system but for small to intermediate levels of noise, partial synchrony is still observable as is shown in Fig. S5.

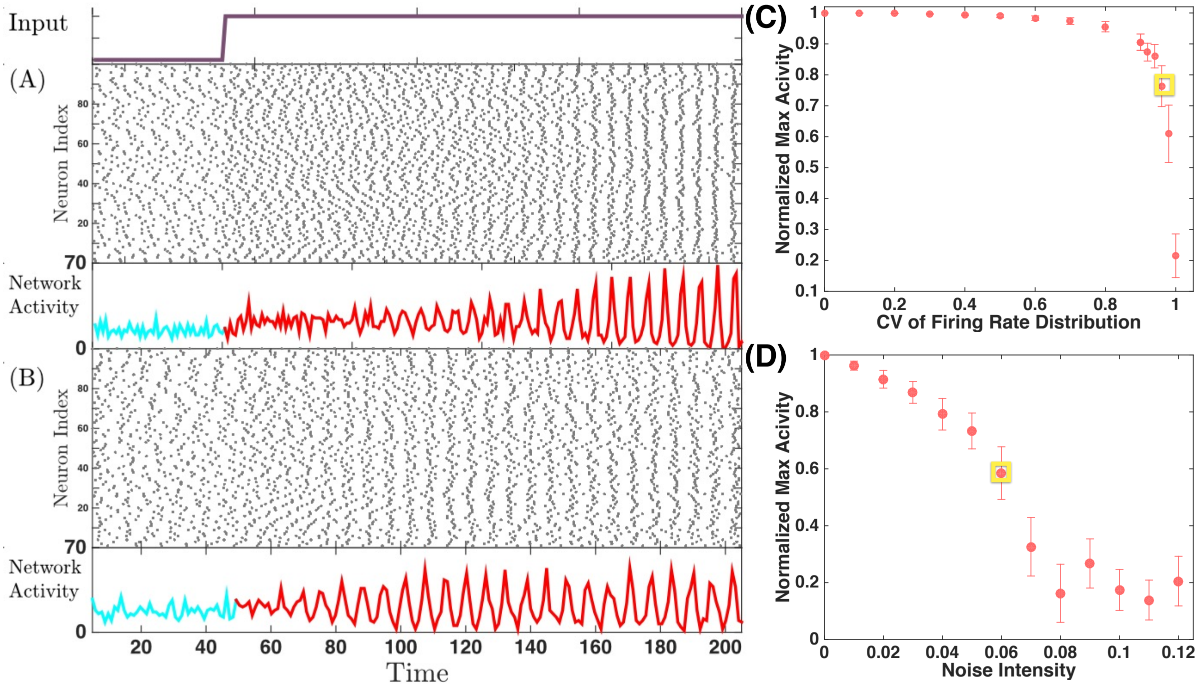

Figure S5: **Transition to synchrony in presence of noise and irregularity** (A) Network activity and raster plot of 100 randomly selected neurons from an all-to-all network ($N = 500$) when the firing rate of neurons are chosen from a lognormal distribution with coefficient of variation equal to 0.94. The normalized activity of the network defined as the ratio of the mean maximum activity in the steady state to the network size, is shown in (C) for different values of the coefficient of variation of firing rates. (B) Network activity and raster plot when an additive white gaussian noise with zero mean is added to the input current. The intensity of the noise $\sqrt{2D}\xi(t)$ is D and here $D = 0.06$. In (D) the mean normalized maximum network activity is shown versus noise intensity. The plot on the top-left shows the level of the mean input which is adjusted to change the network state from incoherent to synchronized.

References

[1] Sadeghi, S. & Valizadeh, A. Synchronization of delayed coupled neurons in presence of inhomogeneity. *Journal of computational neuroscience* **36**, 55–66 (2014).

[2] Esfahani, Z. G. & Valizadeh, A. Zero-lag synchronization despite inhomogeneities in a relay system. *PloS one* **9**, e112688 (2014).

[3] Ota, K. *et al.* Measurement of infinitesimal phase response curves from noisy real neurons. *Physical Review E* **84**, 041902 (2011).
